# Effect of fluid and dietary sodium restriction in the management of patients with heart failure and preserved ejection fraction: study protocol for a randomized controlled trial

**DOI:** 10.1186/1745-6215-15-347

**Published:** 2014-09-04

**Authors:** Karina SM d’Almeida, Eneida R Rabelo-Silva, Gabriela C Souza, Melina M Trojahn, Sofia LS Barilli, Jessica V Mansson, Andreia Biolo, Luis EP Rohde, Nadine Clausell, Luís Beck-da-Silva

**Affiliations:** Graduate Program in Health Sciences, Cardiology and Cardiovascular Sciences, Universidade Federal do Rio Grande do Sul, Rua Ramiro Barcelos, 200, 90035-003 Porto Alegre, RS Brazil; Nutrition Program, Universidade Federal do Pampa, UNIPAMPA, Rua Luiz Joaquim de Sá Britto, s/n, 97650-000 Itaqui, RS Brazil; Serviço de Cardiologia, Heart Failure and Transplant Group, Hospital de Clínicas de Porto Alegre, Rua Ramiro Barcelos, 2350, Sala 2060, 90035-903 Porto Alegre, RS Brazil; School of Nursing, Universidade Federal do Rio Grande do Sul, Rua São Manoel, 963, 90620-110 Porto Alegre, RS Brazil; Department of Internal Medicine, School of Medicine, Universidade Federal do Rio Grande do Sul, Rua Ramiro Barcelos, 2400, 90035-003 Porto Alegre, RS Brazil

**Keywords:** Heart failure, Dietary sodium, Preserved ejection fraction, Randomized clinical trial

## Abstract

**Background:**

Although half of all patients with heart failure (HF) have a normal or near-normal ejection fraction and their prognosis differs little from that of patients with a reduced ejection fraction, the pathophysiology of HF with preserved ejection fraction (HF-PEF) is still poorly understood, and its management poorly supported by clinical trials. Sodium and fluid restriction is the most common self-care measure prescribed to HF patients for management of congestive episodes. However, its role in the treatment of HF-PEF remains unclear. This trial seeks to compare the effects of a sodium- and fluid-restricted diet versus an unrestricted diet on weight loss, neurohormonal activation, and clinical stability in patients admitted for decompensated HF-PEF.

**Methods/Design:**

This is a randomized, parallel trial with blinded outcome assessment. The sample will include adult patients (aged ≥18 years) with a diagnosis of HF-PEF admitted for HF decompensation. The patients will be randomized to receive a diet with sodium and fluid intake restricted to 0.8 g/day and 800 mL/day respectively (intervention group) or an unrestricted diet, with 4 g/day sodium and unlimited fluid intake (control group), and followed for 7 days or until hospital discharge. The primary outcome shall consist of weight loss at 7 days or discharge. The secondary outcome includes assessment of clinical stability, neurohormonal activation, daily perception of thirst and readmission rate at 30 days.

**Discussion:**

Assessment of the effects of sodium and fluid restriction on neurohormonal activation and clinical course of HF-PEF can promote a deeper understanding of the pathophysiology and progression of this complex syndrome.

**Trial registration number:**

ClinicalTrials.gov identifier: NCT01896908 (date of registration: 8 August 2013).

## Background

Heart failure (HF) with preserved ejection fraction (HF-PEF), historically known as diastolic HF, is the clinical syndrome characterized by HF with normal or near-normal systolic function [1]. More recent epidemiological studies and registries have suggested that HF with reduced ejection fraction and HF-PEF are equally prevalent, with the latter accounting for 40 to 71% (mean approximately 50%) of cases [2, 3].

The etiology and epidemiology of HF-PEF appear to be distinct from those of HF with reduced ejection fraction. Women and older adults with comorbidities such as hypertension, diabetes, obesity, and coronary artery disease are more commonly affected [4–8].

Despite the importance of HF-PEF, the pathophysiology and treatment of this phenotype of HF are still poorly understood. Thus far, the major clinical trials (I-Preserve, PEP-CHF, CHARM-Preserved, DIG-CHF and TOPCAT trial) [9–13] have failed to demonstrate the efficacy of any specific treatment on mortality in these patients.

Currently, the mainstay of HF-PEF treatment is symptomatic care and management of comorbidities that predispose patients to this condition, including hypertension, diabetes, ischemia, and arrhythmias [14, 15], and include guidance on lifestyle modifications, such as restricted sodium and fluid intake [14, 15]. Sodium restriction is the self-care measure most commonly prescribed to patients with HF, as it appears to be effective in reducing congestion. However, the evidence base for this recommendation is scarce, and the few studies that have been conducted have yielded inconsistent findings [16–25]. Research has focused on the physiological effects of intake of different amounts of sodium, and few have assessed morbidity and mortality outcomes [17–25]. Except for an observational study by Lennie and colleagues [24] that compared number of hospital admissions and emergency department visits between patients with a sodium intake of >3 g/day versus <3 g/day, no studies have evaluated the effects of sodium restriction in patients with HF-PEF; therefore, its role in these patients remains unknown. Recently, however, Hummel and colleagues [26], investigating the effects of the sodium restricted DASH diet, showed an increased urinary aldosterone excretion during low-sodium diet in patients with HF-PEF.

In an attempt to bridge the knowledge gap in patients with decompensated HF-PEF, this trial seeks to compare the effect of a sodium- and fluid-restricted diet versus an unrestricted diet on weight loss, neurohormonal activation, and clinical stability at 7 days or at hospital discharge in patients admitted for decompensation of HF-PEF.

## Methods/Design

### Study design and centers

This is a randomized, parallel trial with blinded outcome assessment. The study population will comprise patients with a diagnosis of HF-PEF who have presented to hospital with HF decompensation. The study will be carried out at Hospital de Clínicas de Porto Alegre (HCPA), Brazil, and take place at the Emergency Department or other inpatient units.

### Inclusion and exclusion criteria

The sample will include adult patients with a diagnosis of HF (and left ventricular ejection fraction >50%) within 36 hours of hospital admission for decompensation of HF – defined as clinical signs of congestion, history of dyspnea and orthopnea in the week before hospitalization, and B-type natriuretic peptide (BNP) levels >100 pg/mL – who agree to take part in the study and provide written informed consent. Patients with an estimated glomerular filtration rate (Modification of Diet in Renal Disease formula) ≤30 mL/minute, those with cardiogenic shock, HF due to valvular disease and those whose survival is jeopardized by another ongoing condition and/or by difficulty adhering to treatment (for example, due to dementia or cognitive deficits) will be excluded.

### Ethical considerations

All procedures were conducted in accordance with the ethical standards for human subject research set forth in the Declaration of Helsinki. Written informed consent shall be obtained from all patients included in the trial. The project was approved by the Research Ethics Committee of Hospital de Clínicas de Porto Alegre (registration number 12–0437) and is registered in the ClinicalTrials.gov database under identification number NCT01896908.

### Sample size

Sample size calculations were carried out in WINPEPI 11.20 (Brixton Health, Israel). We used the weight loss values reported in the studies of Aliti and colleagues [25] and Cardoso and colleagues [27], which assessed weight loss in patients with HF and systolic dysfunction admitted for HF decompensation and congestion. For a significance level of α = 0.05 and a statistical power of 80%, with a standard deviation of 3 kg and a between-group difference in weight corresponding to a weight loss of 2 kg in 7 days, the minimum sample size will be 74 patients (37 randomized to each group).

### Interventions

The control group (CG) will receive the standard hospital diet, which provides approximately 4 g sodium (10 g salt)/day and unlimited fluid intake. The intervention group (IG) will receive an otherwise identical diet restricted to 0.8 g sodium (2 g salt)/day and 800 mL fluids/day until the 7th day of admission or hospital discharge, whichever comes first.

### Study protocol

Patients who meet the inclusion criteria and are eligible will be invited to take part in this trial during their hospitalization. Data will be collected on sociodemographic parameters, clinical variables, results of laboratory tests performed as part of routine patient care (creatinine, urea, potassium, sodium, complete blood count, and BNP on admission), current medications, body weight, and clinical congestion score, and blood will be collected for assessment of neurohormonal activation. Patients will then be randomized to IG or CG.

Once allocation has been completed, the on-call dietitian will be notified so as to change the participants’ meal plan as appropriate. The same dietary prescription will be used for participants in both the IG and CG: *DIET AS PER RESEARCH PROTOCOL. PATIENT WILL RECEIVE DIET UNTIL __/__ OR DISCHARGE. PLEASE DO NOT ALTER DIET.* This practice will be implemented in agreement with the medical staff and with the hospital Department of Nutrition and Dietetics.

A daily check of medical records will be performed to identify modification of any drug prescription. The search for hypotensive episodes will be performed by checking blood pressure every 6 hours and by questioning patients about symptoms of hypotension (dizziness, fainting or lightheadedness) on a daily basis.

**Figure 1:**
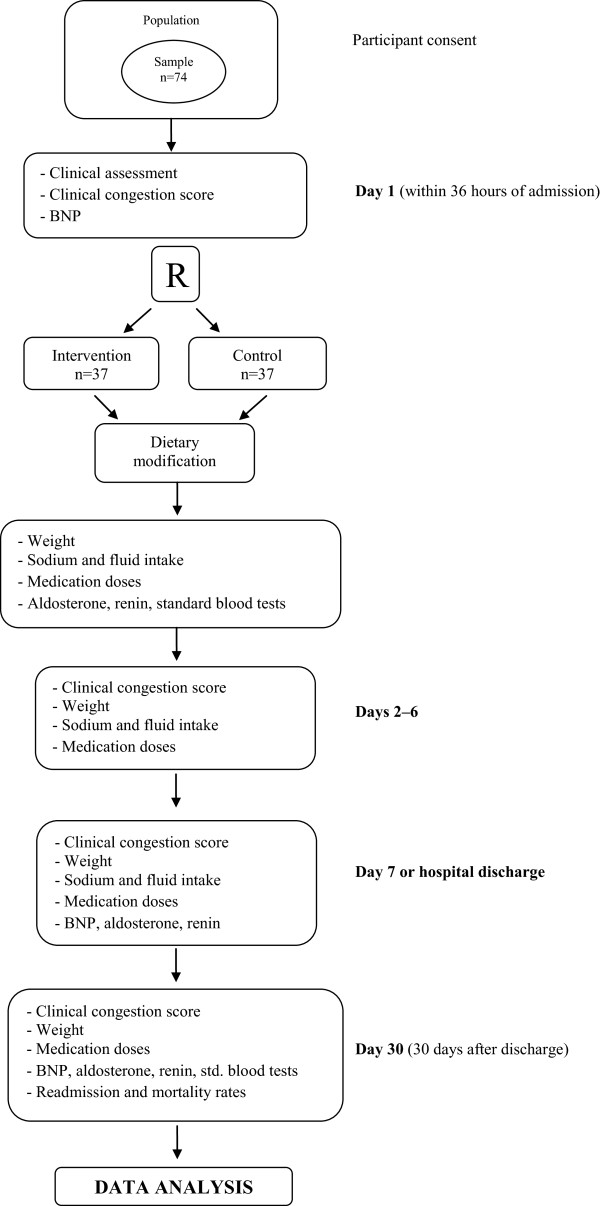
**CONSORT flowchart of the study.** BNP, B-type natriuretic peptide; R, randomization; std., standard.

Outcomes assessment 30 days after discharge will be carried out at the study facility by means of a face-to-face encounter between the investigators and each participant, at which time a clinical assessment will be performed and blood will be collected for analysis of neurohormonal activation (Figure 1).

### Randomization

The randomization will be performed through a simple sequential randomization plan generated online, using the http://www.randomization.com website.

### Blinding

During hospitalization, the medical staff involved in patient care will be blind to group allocation. All clinical assessments during hospitalization and at 30-day follow-up will be conducted by a nurse also blinded to group allocation.

### Variables in the study

#### Clinical variables

##### Clinical congestion score

The clinical congestion score is an instrument composed of seven questions designed to assess signs and symptoms of congestion, including presence of pulmonary crackles, third heart sound, jugular venous distension, peripheral edema, hepatojugular reflux, orthopnea, paroxysmal nocturnal dyspnea, and New York Heart Association functional class. This score ranges from 1 to 22 points, with higher scores being directly indicative of worse congestion [28].

##### Blood pressure and heart rate

Blood pressure and heart rate will be measured by the trial investigators shortly after randomization.

##### Estimation of glomerular filtration rate

The Modification of Diet in Renal Disease formula will be used for estimation of the glomerular filtration rate [29].

##### Left ventricular ejection fraction

The left ventricular ejection fraction will be assessed by means of echocardiography, using the Teichholz method or, if available, the Simpson method.

##### Body weight

Weight will be measured with participants barefoot, wearing minimal clothing, and standing on the center of a digital platform scale, and recorded in a spreadsheet.

##### Medical history

Data on the etiology of HF, history of present illness, past medical history, comorbidities, and current medications will be collected from patient records and checked during the patient interview.

### Demographic variables

A structured questionnaire will be administered to all participants for collection of demographic parameters (age, sex, ethnicity), socioeconomic, and educational data.

### Laboratory variables

Blood samples will be collected by a trained professional at the time of study enrollment, at hospital discharge, and 30 days after discharge. Samples will be centrifuged at 4°C, 3,670 rpm, for 10 minutes and stored in Eppendorf tubes at -80°C for later analysis of neurohormonal activation (renin and aldosterone).

#### General blood work

General blood work will comprise of complete blood count, urea (ultraviolet kinetic assay), serum creatinine (Jaffé method), plasma sodium and potassium (ion selective electrode).

#### B-type (brain) natriuretic peptide

Immunofluorescence methods will be used for quantitation of BNP in plasma.

#### Renin

A radioimmunoassay for measurement of angiotensin I generated by the action of renin on its substrate, angiotensinogen, will be used for quantitation of plasma renin activity.

#### Aldosterone

A solid phase radioimmunoassay based on aldosterone-specific antibodies will be used for quantitation of serum aldosterone levels.

### Primary outcome

The primary outcome measure shall consist of weight loss at 7 days or discharge.

### Secondary outcomes

The secondary outcome measures shall consist of:

- Clinical stability, defined as improvement in symptoms with no evidence of congestion (assessed by congestion score); weight stable for 2 days, with no loss or gain greater than 1 kg (daily weight check); no intravenous HF drugs for 48 hours (daily medication record: diuretics, vasodilators); no increase in diuretics dose for 48 hours (daily medication record).
- Neurohormonal activation: assessment of neurohormonal activation shall include measurement of serum renin, aldosterone, and BNP levels on admission, at discharge, and at 30 days.
- Daily perception of thirst: a visual scale (with values ranging from 0 to 10) will be used daily to verify the degree of thirst.
- Hospital readmission rate for all-causes. Patients shall be followed for 30 days after discharge.

### Statistical analyses

Continuous variables following a normal distribution will be expressed as mean ± standard deviation; asymmetrically distributed continuous variables will be expressed as median and interquartile range; and categorical variables will be expressed as absolute and relative frequencies. For between-group comparisons, Student’s *t-*test will be used for normally distributed variables, and the Mann–Whitney *U* test for asymmetrically distributed variables. A paired *t*-test will be used for within-group analysis of body weight and congestion score. The chi-square or Fisher’s exact tests will be used to evaluate associations between categorical variables. The generalized estimating equations test with Bonferroni adjustment will be used for comparison between variables during the study period. The significance level will be set at 5%, and all data will be analyzed in SPSS 18.0 (SPSS Inc., Chicago, IL, USA).

For assessment of clinical stability, in accordance with pre-established criteria, two blinded examiners (physician HF specialists not involved in the care of the study participants) will analyze the outcome variables in both groups.

## Discussion

During protocol, patients will receive the standard hospital diet for both the control (normal diet) and intervention group (low-sodium diet), modified only in the amount of fluid offered to the intervention group. During the study, patients will be instructed to consume only foods and beverages offered by the hospital. The plate-waste method shall be used to measure sodium intake for 7 days or until hospital discharge, whichever comes first. A trained investigator will assess dietary intake twice daily: after lunch, for collection of information on intake during breakfast, morning snack, and lunch; and after dinner, for collection of information on intake during the afternoon snack, dinner, and supper. In addition, a visual scale (with values ranging from 0 to 10) will be used to verify the degree of thirst.

## Trial status

The trial is ongoing. Thirteen patients have completed the study protocol and additional patients are being recruited.

## Abbreviations

BNP: B-type natriuretic peptide
CG: control group
HF: heart failure
HF-PEF: heart failure with preserved ejection fraction
IG: intervention group
